# Current News

**Published:** 1901-04

**Authors:** 


### Current News.

### COMMUNICATIONS FROM THE FOREIGN RELATIONS COMMITTEE.

Department of State,
Washington, February 6, 1901.

W. C. Barrett, Esq.,
*Chairman Committee on Foreign Relations of the National Association of Dental Faculties,* 208 Franklin Street, Buffalo, N. Y.:

Sir,—At the suggestion of the Consul at Munich, I enclose for your information copy of a despatch from the Consul in regard to American dental degrees in Germany, and the efforts which are being made to prevent those who hold fraudulent diplomas from practising dentistry.

I am, sir, your obedient servant,

(Signed) THOS. W. CRILLER,
*Third Assistant Secretary.*

(Copy.)

No. 38.

CONSULATE OF THE UNITED STATES OF AMERICA,
MUNICH, GERMANY, December 29, 1900.

HONORABLE DAVID J. HILL, etc., etc., etc.:

SIR,—Referring most respectfully to my unnumbered despatch of April 21, 1900, upon the subject of American dental degrees in Germany, to which I was honored with a reply by your department under date of July 17, 1900, No. 36, I have the honor most respectfully to report at this time:

1. That I have since placed myself in relation with the organized associations of American dental graduates in Southern Germany, and, in connection with the learned counsel of this Consulate, have advised them how to conduct themselves in their relations with the government and press, and in the defence of those of their members who have been or are being prosecuted for what is termed here an "unlawful" use of their honestly acquired titles of D.D.S.

2. That at the same time, in all cases, whether of gentlemen holding legitimate diplomas or of persons holding illegal issues, I have been in constant communication with the Bavarian Department of Justice and the Foreign Office to protect the rights of all legitimate holders of such American degrees, correctly issued, to use and advertise their degrees, and to secure the prosecution and conviction of those illegally holding American certificates or honors.

My task has been a peculiarly difficult and delicate one, as there is, in the first place, even among educated and intelligent Germans, a misconception of the character of American universities, and especially the schools of dentistry, on account of many of them being, so far as their original organization is concerned, in form at least, private concerns; and among the less informed a strong prejudice against American degrees on this account. It has, therefore, been a matter of propaganda to bring the authorities to understand that under the republican forms of government existing in the several States, where so much is necessarily left to private initiative, these institutions, although in form private enterprises, by virtue of their charters and the right of visitation and control by the State authorities, are in fact public institutions.

Another difficulty lies in the fact that the German universities, stimulated by the reputation and success of American dental colleges, have added dental departments to their curricula, which, in theory at least, are not inferior to the average American institutions; and among others the University of Munich has recently established such a department, which in equipment and the character of its instruction will prove inferior to no other.

The purpose of this instruction in dentistry at the German universities is to offer to Germans the opportunity of educating themselves thoroughly in that art and to raise the estimation of German dental degrees to the American standard, so as to induce students to remain at home.

It is easy to comprehend how this jealousy of American degrees finds its expression not only among prejudiced people, but also among holders of German dental degrees in denunciation of American degrees and dental institutions, and also in efforts to bring about a prohibition of their use in Germany.

I have good reason to believe that I have met these difficulties successfully and have been able to convince the authorities here of the value of legitimate American university honors and the titles of technical schools, and of the expediency of not prohibiting them; also of the sincere desire of the United States government to do everything possible to prevent the issue of worthless diplomas, and to effect the closing of institutions issuing them.

My main endeavor has been to secure such evidence as might be of service in proceedings against the institutions issuing illegitimate diplomas, and I have already obtained possession of original diplomas and certificates in two instances where they were purchased in America by Germans, against whom proceedings are now pending.

In one of these cases I have had the diplomas copied by photography and type-written copies of the certificates made, specimens of which are hereto annexed marked Exhibits A, B, C, D, E, F. I have applied to the legal authorities to have the original diplomas and certificates in these cases delivered to me for transmission to the State Department, for use as evidence in any proceedings it may be deemed expedient to institute, and though such a course is difficult to effect, I hope for a favorable answer.

Owing to the urgency of the case, I have also transmitted copies of these diplomas and certificates to his Excellency the Governor of Illinois and a copy of my letter to him is hereto annexed, marked Exhibit G.

On December 10, 1900, a very interesting case was settled in the courts of Munich against one Samuel Gumpoldt, once a "Zahntechniker," now a full-fledged "American Dentist," claiming also to be the holder of the American degree of Doctor of Dental Surgery. He obtained the "Doctorate" at one of those non-reputable dental schools, of which two remain to be suppressed in Chicago. "Dr." Gumpoldt went to America some time last spring, remaining a few weeks in Chicago, and came back with a certificate from the "State Board of Dental Examiners," permitting him to practise dentistry in Illinois. The States Attorney here made the polite request that I should testify as an expert in the case in order to establish the illegality of the defendant's claim, and as a result the "Dr." was condemned for terming himself "Amerikanischer Zahnarzt" and heavily fined. The case, of course, will be appealed, but it is to be hoped that the governor of Illinois will cause an inquiry into the illegal practice prevailing in that State by issuing such certificates as in this instance, as the State Board is only expected to admit to examination a candidate who has spent at least six months in a regular dental school. In this instance the "Dr." made certain claims as to studies in Roumania, but I fear the State Board of Illinois has no "evidence" to substantiate these claims.

Another case now in the courts is affording me the opportunity to secure by the aid of photography the needful evidence to convict of such illegal practice the other now remaining non-reputable institution in Illinois making a business of the sale of diplomas, and I shall have the honor to submit this report by an early post.

The rapidly growing tendency among the peoples of the German Empire to bar out as far as possible all foreign competition may, as I have already suggested, force the governments of the various States to a more determined warfare in behalf of the dentists educated in the schools of Germany only, against those bearing the distinctive honors of the American dental schools, thus ultimately affecting not only the good standing of American dentists abroad, but also destroying their usefulness, if not barring them out altogether. It is to be hoped, therefore, that the course taken by this Consulate, however great the sacrifice in labor and time, may prove both timely and judicious, maintain the integrity of our worthy schools of dentistry, and preserve them in honor abroad as well as at home.

To this end I would most respectfully ask you whether you do not deem it expedient that publicity be given through the press in America to such institutions and in Germany to punish persons holding and advertising their diplomas, in order to deter foreigners from purchasing such titles and thereby to destroy the market for them. I have abstained entirely from any communications whatever to the press, but believe that the widest publicity should be given the whole subject.

I have the honor, etc.,

(Signed) James H. Worman,
*United States Consul.*

(Enclosures.)

Photograph of diploma granted "Johannes Fuchs," conferring the degree of Doctor of Dental Surgery, granted by the "Cosmopolitan Post-Graduate College" of Chicago. Dated October 25, 1899. Signed, C. A. Weil, Dr. Med., Chancellor; Emanuel Kargan, D.D.S., Dean; C. A. Williams, Rector.

Photograph of diploma granted Dr. Johannes Fuchs by the Haskell Post-Graduate School of Prosthetic Dentistry. Dated October 16, 1899. Signed, L. P. Haskell, Pres.; G. A. Grant, Secretary.

(Copy.)

Buffalo, N. Y., February 9, 1901.

Department of State.

Thos. W. Criller, *Assistant Secretary:*

Sir,—I am in receipt of a communication from you enclosing one from the United States Consul at Munich, concerning the American dental diploma and other matters. I can assure you, sir, that I deem it of the very highest importance to the dental profession of America, and I believe that the Consul is doing a work that will advance the interests of many American citizens. Unless I am advised that the communications are in any way confidential, I shall forward them for publication in some of our most important professional journals. I beg that any future advices that the State Department may receive of the same nature may be forwarded to me for communication to the dental profession of America.

I am very truly yours,

(Signed) W. C. Barrett,

*Chairman Foreign Relations Committee, National Association American Dental Colleges.*

Department of State,
Washington, February 13, 1901.

W. C. Barrett,

*Chairman Foreign Relations Committee, National Association Dental Faculties,* Buffalo, N. Y.:

Sir,—I have to acknowledge the receipt of your letter of the 9th inst., suggesting that the despatch from the Consul at Munich in regard to bogus dental institutions be published. In reply I have to say that the Department sees no objection to your publishing it.

I am, sir, your obedient servant,

David J. Hill,
*Acting Secretary.*

(Copy.)

Buffalo, N. Y., February 9, 1901.

Hon. James H. Worman,

*United States Consul at Munich:*

Sir,—I am in receipt of a communication from the Department of State of the United States government enclosing a report, or copy of a despatch, concerning American dental degrees in Germany. I enclose copy of my answer to the Third Assistant Secretary in reference thereto. I beg personally to assure you that the great number of dentists in the United States and the graduates of American colleges abroad will fully appreciate the importance of the work you have undertaken, and will extend to you their enthusiastic support.

By this mail I will forward to you copies of the reports of this committee, which will give you some idea of what we are attempting to do. Let me say that we now have in prison a number of those who have been engaged in issuing fraudulent diplomas, and hope the traffic is now upon the point of being broken up in America.

I beg, on the part of my colleagues, to tender any service on our part in your good work, and I request that I may be favored with any reports or other documents which may assist in the work in which we are engaged.

Very truly yours,

(Signed) W. C. Barrett,

*Chairman Foreign Relations Committee, National Association American Dental Colleges.*

---

### THE FIFTH DISTRICT DENTAL SOCIETY.

The Fifth District Dental Society of the State of New York is to tender a complimentary dinner to Dr. S. B. Palmer, of Syracuse, N. Y., in honor of his long years of faithful service, both in his office and in behalf of the profession at large.

Representative members of the dental fraternity will be present, and the affair promises to be most enjoyable.

The dinner is to be given at Syracuse, on the evening of April 13, 1901, and an invitation is extended to the profession to be present. Dinner-tickets will be issued before April 1, on receipt of $5.00. Address the chairman of the committee in charge, Dr. G. B. Beach, 518 S. A. & K. Building, Syracuse, N. Y.

G. B. Beach,
A. Retter,
Sheridan Slocum,
G. H. Butler,
J. C. Curtis,
*Committee.*

---

### TRI-STATE DENTAL ASSOCIATION.

The Tri-State Dental Association (Indiana, Kentucky, and Illinois) meets in Paducah, Ky., May 28, 29, and 30, 1901. W. H. Brosman, Secretary, Albion, Ill.; W. H. Pitcher, President, Paducah, Ky.

W. H. Brosman,
*Secretary.*

